# A phase Ib study of camrelizumab in combination with apatinib and fuzuloparib in patients with recurrent or metastatic triple-negative breast cancer

**DOI:** 10.1186/s12916-022-02527-6

**Published:** 2022-10-03

**Authors:** Qingyuan Zhang, Bin Shao, Zhongsheng Tong, Quchang Ouyang, Yuting Wang, Guoying Xu, Shaorong Li, Huiping Li

**Affiliations:** 1 Department of Breast Oncology, Harbin Medical University Cancer Hospital, Harbin, China; 2 Department of Breast Oncology, Key Laboratory of Carcinogenesis and Translational Research (Ministry of Education/Beijing), Peking University Cancer Hospital and Institute, No.52 Fucheng Road, Haidian District, Beijing, 100142 China; 3 Department of Breast Oncology, Tianjin Medical University Cancer Institute and Hospital, Tianjin, China; 4 Department of Breast Oncology, Hunan Cancer Hospital, Changsha, China; 5 Clinical Research & Development, Jiangsu Hengrui Pharmaceuticals Co. Ltd., Shanghai, China

**Keywords:** Camrelizumab, Apatinib, Fuzuloparib, TNBC, PARP, VEGFR, PD-1, Triple-negative breast cancer, Immunotherapy

## Abstract

**Background:**

Strategies to improve activity of immune checkpoint inhibitors for triple-negative breast cancer (TNBC) are needed. Preclinical studies showed that antiangiogenic agents and poly (ADP-ribose) polymerase (PARP) inhibitors might sensitize tumors to immunotherapy. Here, we investigated the tolerability, safety, and preliminary antitumor activity of camrelizumab, an anti-PD-1 antibody, in combination with apatinib, a vascular endothelial growth factor receptor-2 inhibitor, and fuzuloparib, a PARP inhibitor, in patients with recurrent or metastatic TNBC.

**Methods:**

This phase Ib study included a dose-finding part and a dose-expansion part. In the dose-finding part, a 3 + 3 dose escalation scheme was introduced. Patients were given camrelizumab (200 mg every 2 weeks) plus apatinib (375 mg or 500 mg once daily) and fuzuloparib (starting dose 100 mg twice daily) every 28-day cycle. After evaluation of the tolerability and safety of the dosing regimens, a clinical recommended dose was determined for the dose-expansion part. The primary endpoint was dose-limiting toxicity (DLT).

**Results:**

A total of 32 patients were enrolled. Three patients received camrelizumab 200 mg + apatinib 375 mg + fuzuloparib 100 mg, and 29 received camrelizumab 200 mg + apatinib 500 mg + fuzuloparib 100 mg (clinical recommended dose). No DLTs were observed in either group. The most common grade ≥ 3 treatment-related adverse events were decreased white blood cell count (20.7%), hypertension (13.8%), decreased neutrophil count (10.3%), and increased aspartate aminotransferase (10.3%). Two patients discontinued study treatment due to immune-mediated hepatitis (*n* = 1) and anemia, decreased platelet count, decreased white blood cell count, increased alanine aminotransferase, increased aspartate aminotransferase, and increased γ-glutamyltransferase (*n* = 1). One patient died of unknown cause. Two (6.9% [95% CI, 0.9–22.8]) of 29 patients with camrelizumab 200 mg + apatinib 500 mg + fuzuloparib 100 mg had objective response. The disease control rate was 62.1% (95% CI, 42.3–79.3). The median progression-free survival was 5.2 months (95% CI, 3.6–7.3), and the 12-month overall survival rate was 64.2% (95% CI, 19.0–88.8).

**Conclusions:**

Combination of camrelizumab plus apatinib and fuzuloparib showed manageable safety profile and preliminary antitumor activity in patients with recurrent or metastatic TNBC.

**Trial registration:**

ClinicalTrials.gov NCT03945604 (May 10, 2019).

**Supplementary Information:**

The online version contains supplementary material available at 10.1186/s12916-022-02527-6.

## Background

Triple-negative breast cancer (TNBC), which is characterized by negative expression of the estrogen receptor (ER), progesterone receptor (PR), and human epidermal growth factor receptor 2 (HER2), accounts for approximately 15% of all breast cancers [1]. TNBC is an aggressive disease with higher rates of recurrence and metastasis than other breast cancer subtypes [2].

Due to the lack of molecular biomarkers in TNBC, taxane- and anthracycline-based chemotherapy remains the major standard-of-care in the (neo)-adjuvant, recurrent or metastatic setting [3, 4]. New agents with better therapeutic effects are greatly needed. The introduction of immunotherapy has changed the treatment paradigm in many cancer types [5]. However, immune checkpoint inhibitors alone fail to prolong overall survival (OS) compared with chemotherapy in previously treated metastatic TNBC [6]. Tumor response to immune checkpoint inhibitors depends on the tumor microenvironment, and tumor angiogenesis contributes to the construction of an immunosuppressive microenvironment by decreasing the abundance and function of antitumor lymphocytes, inhibiting dendritic cell maturation, and activating regulatory T cells [7–10]. Therefore, it is hypothesized that inhibition of angiogenesis may sensitize tumors to immunotherapy, and addition of antiangiogenic agents to programmed cell death-1 (PD-1) inhibitors could result in an improved antitumor response in breast cancer [11, 12]. A previous study showed that the combination of camrelizumab (a humanized anti-PD-1 antibody) and apatinib (an oral vascular endothelial growth factor receptor-2 [VEGFR-2] tyrosine kinase inhibitor) demonstrated a favorable objective response rate (ORR) compared with either as monotherapy in patients with advanced TNBC [13].

Poly (ADP-ribose) polymerase-1 (PARP-1) is a central enzyme that functions in the repair of DNA single-strand breaks [14]. Breast cancers with germline *BRCA1* and/or *BRCA2* mutations have defects in the repair of DNA double-strand breaks by means of homologous recombination and are sensitive to PARP inhibitors through synthetic lethality [15–17]. TNBC shares similar gene expression characteristics with tumors derived from germline *BRCA1* mutation carriers, and deficiency in DNA damage repair is considered a hallmark of some triple-negative tumors [18–20]. However, PARP inhibitor monotherapy has shown no evidence of benefit in patients with late-stage metastatic TNBC [21]. On the basis of the evidence above, we speculated that combining immune checkpoint inhibitor with antiangiogenic agent and PARP inhibitor might have a synergistic effect for the treatment of TNBC. Here, we report the tolerability, safety, preliminary antitumor activity, and pharmacokinetics of camrelizumab in combination with apatinib and fuzuloparib, an orally active PARP inhibitor, in patients with recurrent or metastatic TNBC.

## Methods

### Study design and patients

This open-label, multicenter, dose-finding and dose-expansion phase Ib study was conducted to investigate the tolerability, safety, preliminary antitumor activity, and pharmacokinetics of the combination therapy in patients with recurrent or metastatic TNBC (ClinicalTrials.gov identifier NCT03945604). Eligible patients were female and 18 years of age or older and had histologically confirmed recurrent or metastatic TNBC (defined as lack of ER and PR expression by immunohistochemistry [IHC positive tumor cells < 1%] and HER2 negativity [IHC 0/1 + , or IHC 2 + but negative by fluorescence in situ hybridization/chromogenic in situ hybridization]). Patients had received previous taxane- or anthracycline-based chemotherapy in the (neo)-adjuvant, recurrent, or metastatic setting and had disease progression during or after systemic treatment in the recurrent or metastatic setting. Patients who had received taxane- or anthracycline-based chemotherapy in the (neo)-adjuvant setting and refused to receive chemotherapy for recurrent or metastatic disease were allowed. No more than 2 prior lines of chemotherapy in the recurrent or metastatic setting were permitted. Previous platinum-based chemotherapy for recurrent or metastatic disease was allowed if at least 4 cycles had been administered, with clear evidence of tumor stability or regression during treatment. Additional inclusion criteria included an Eastern Cooperative Oncology Group (ECOG) performance status of 0 or 1, at least one measurable lesion according to Response Evaluation Criteria in Solid Tumors (RECIST) version 1.1, a life expectancy of at least 12 weeks, and adequate organ function. Exclusion criteria were active or history of autoimmune disease; use of systemic immunosuppressive agents within 4 weeks before study entry; allergy to other monoclonal antibodies; untreated active brain metastases; prior treatment with anti-PD-1 antibody, anti-PD-L1 antibody, apatinib, or PARP inhibitors; completion of prior chemotherapy or surgery within the previous 4 weeks, palliative radiotherapy within the previous 2 weeks, or oral targeted therapy within 5 half-lives before study entry, or adverse events from previous therapy that had not resolved to grade ≤ 1.

The trial was performed in accordance with the Declaration of Helsinki and the International Council for Harmonization Good Clinical Practice. The study protocol was approved by the independent ethics committee at each site. All patients provided written informed consent before enrollment.

### Treatment and assessments

This study included a dose-finding part and a dose-expansion part. In the dose-finding part, a 3 + 3 dose escalation scheme was introduced. Patients were given camrelizumab (fixed dose 200 mg intravenously every 2 weeks) plus apatinib (starting dose 375 mg orally once daily) and fuzuloparib (starting dose 100 mg orally twice daily) every 28-day cycle. If two or more out of the first three to six patients experienced dose-limiting toxicity (DLT) during cycle 1, dose de-escalation of fuzuloparib (to 80 mg twice daily) or apatinib (to 250 mg once daily) was planned after taking into consideration of the DLT and apatinib plasma concentration observed in the first cycle. If fewer than two of six patients experienced DLT during cycle 1, this dosage was considered tolerable, and the decision of whether to escalate the dose of apatinib to 500 mg once daily was made on the basis of the DLT, safety, and apatinib plasma concentration seen during the first cycle. After comprehensive evaluation of the tolerability and safety of the dosing regimens, a clinical recommended dose was determined for the dose-expansion part. In the dose-expansion part, 21 patients were administered the recommended dose. If four or more responders among the 21 patients were identified by imaging assessment, enrollment would continue to 34 patients. Treatment continued until disease progression, intolerable adverse events, start of new antitumor treatment, withdrawal of consent, or loss to follow-up.

Adverse events were monitored throughout the study treatment from informed consent until 90 days after the last camrelizumab dose or 30 days after the last apatinib or fuzuloparib dose, whichever occurred later, and were graded according to the National Cancer Institute Common Terminology Criteria for Adverse Events version 5.0. Radiographic assessment was performed by computed tomography (CT) or magnetic resonance imaging (MRI) according to RECIST version 1.1 at baseline and every two cycles. Complete or partial responses were confirmed 4 weeks later. Blood samples for pharmacokinetic analysis of apatinib and fuzuloparib were collected in all patients enrolled in the dose-finding part and in six to eight patients enrolled in the dose-expansion part. To evaluate the pharmacokinetic data of single-agent apatinib, patients intended for pharmacokinetic analysis were given apatinib alone 3 days before combination treatment initiation. Blood samples were collected 30 min pre-dose, 30 min, 1 h, 2 h, 3 h, 4 h, 8 h, 12 h, and 24 h post-dose on day − 3 of cycle 0 and day 1 of cycle 1.

### Definition of DLT

DLT was defined as any of the following adverse events related to the study medicines according to investigator opinion and was documented during the first cycle in the dose-finding part: grade 4 hematological toxicities, grade ≥ 3 thrombocytopenia with bleeding, or grade ≥ 3 febrile neutropenia (38.5 °C); grade ≥ 3 non-hematological toxicities (excluding laboratory abnormalities and grade 3 hypertension, rash, diarrhea, nausea and vomiting that were well controlled by treatment); grade ≥ 3 laboratory abnormalities leading to hospitalization lasting 7 days or longer; toxicities resulting in failure to complete 2 doses of camrelizumab during the first cycle or a camrelizumab administration delay of at least 3 days in cycle 2; and toxicities causing cumulative dose interruption of apatinib or fuzuloparib for at least 7 days.

### Endpoints

The primary endpoint was DLT in cycle 1. The secondary endpoints were safety, antitumor activity (ORR, defined as the percentage of patients achieving a confirmed complete response or partial response per RECIST version 1.1; duration of response [DOR, defined as time from the first documented confirmed objective response to progressive disease or death, whichever occurred earlier]; disease control rate [DCR, defined as the percentage of patients with complete response, partial response, and stable disease]; progression-free survival [PFS, defined as the time from treatment initiation to documented progressive disease or death, whichever occurred earlier]; and 12-month OS rate), and pharmacokinetics of apatinib and fuzuloparib, including maximum plasma concentration (*C*_max_), time to *C*_max_ (*t*_max_), and area under the plasma concentration time curve (AUC).

### Statistical analyses

The dose-finding part followed a 3 + 3 scheme. For the dose-expansion part, Simon’s two-stage minimax design was employed. Assuming an unacceptable ORR of 15% and an expected ORR of 35%, with a two-sided alpha of 0.05 and 80% power, 21 patients (including those who received the recommended dose in the dose-finding part) were planned in the first stage. If four or more responses were observed after the first tumor response assessment, the study proceeded to stage 2 and enrollment was expanded to 34 patients (including patients who received the recommended dose in the dose-finding part). The study would be deemed promising if 12 or more responses were observed among the 34 patients. Considering a dropout rate of 15%, a total of 40 patients were required for the dose-expansion part.

Safety and antitumor activity analyses were performed in all patients who received at least one dose of the study treatment. Continuously distributed data were summarized as mean (standard deviation) or median (range). Categorical data were summarized by the number and percentage of patients in each category. Adverse events were summarized descriptively. Two-sided 95% confidence intervals (CIs) for ORR and DCR were calculated using the Clopper-Pearson method. The PFS, DOR, and 12-month OS rate and their corresponding 95% CIs were estimated using the Kaplan–Meier method. Analyses of safety and antitumor activity were performed with SAS version 9.4. Pharmacokinetic parameters were descriptively summarized with non-compartmental analysis using WinNonlin (version 8.2).

## Results

### Patient baseline characteristics and distribution

Between June 04, 2019, and August 25, 2020, a total of 32 patients with recurrent or metastatic TNBC were enrolled, with six patients in the dose-finding part and 26 in the dose-expansion part. In the dose-finding part, three patients each received camrelizumab 200 mg + apatinib 375 mg + fuzuloparib 100 mg and camrelizumab 200 mg + apatinib 500 mg + fuzuloparib 100 mg, respectively, and no DLTs were recorded in either group. Thus, camrelizumab 200 mg + apatinib 500 mg + fuzuloparib 100 mg dosing regimen was recommended for the dose-expansion part. As of data cutoff on February 9, 2021, the median follow-up duration was 7.7 months (range, 0.4–20.0) in all patients. Three patients with camrelizumab + apatinib 500 mg + fuzuloparib remained on study treatment. The baseline characteristics are summarized in Table 1. The median age was 42 years in patients with camrelizumab + apatinib 375 mg + fuzuloparib and 54 years in patients with camrelizumab + apatinib 500 mg + fuzuloparib. Ten (31.3%) of the 32 patients had an ECOG performance score of 1.

**Table 1:** Baseline characteristics

	**Camrelizumab + fuzuloparib + apatinib 375 mg (*n* = 3)**	**Camrelizumab + fuzuloparib + apatinib 500 mg (*n* = 29)**
Age, median (range), years	42 (37–49)	54 (39–72)
ECOG performance status		
0	2 (66.7)	20 (69.0)
1	1 (33.3)	9 (31.0)
Metastasis		
Yes	0	4 (13.8)
No	3 (100.0)	22 (75.9)
Unknown	0	3 (10.3)
Previous neo-(adjuvant) chemotherapy		
Yes	3 (100)	29 (100)
No	0	0
Previous palliative chemotherapy		
Yes	3 (100.0)	15 (51.7)
No	0	14 (48.3)
No. of prior palliative therapies		
1	2 (66.7)	9 (31.0)
2	0	3 (10.3)
3	1 (33.3)	2 (6.9)
4	0	1 (3.4)
Previous taxane and anthracycline treatment		
Taxane	3 (100.0)	28 (96.6)
Anthracycline	3 (100.0)	27 (93.1)

Data are *n* (%) or otherwise indicated. *ECOG* Eastern Cooperative Oncology Group

### Safety

All patients were evaluable for safety. In the camrelizumab + apatinib 375 mg + fuzuloparib group, the median duration of exposure for camrelizumab was 2.4 months (range, 1.9–3.3), the median duration of exposure for apatinib was 2.1 months (range, 1.9–3.2), and the median duration of exposure for fuzuloparib was 2.1 months (range, 1.9–3.2). In the camrelizumab + apatinib 500 mg + fuzuloparib group, the median duration of exposure was 4.2 months for camrelizumab (range, 0.5–16.7), the median duration of exposure for apatinib was 4.1 months (range, 0.3–16.7), and the median duration of exposure for fuzuloparib was 4.1 months (range, 0.3–16.7). Adverse events of any cause were reported in all patients. Grade ≥ 3 treatment-related adverse events (TRAEs) occurred in one (vomiting) of three patients in the camrelizumab + apatinib 375 mg + fuzuloparib group and 17 (58.6%) of 29 patients in the camrelizumab + apatinib 500 mg + fuzuloparib group. In the camrelizumab + apatinib 500 mg + fuzuloparib group, the most common TRAEs of grade 3 or higher were decreased white blood cell count (20.7%), hypertension (13.8%), decreased neutrophil count (10.3%), and increased aspartate aminotransferase (10.3%) (Table 2). Adverse events leading to discontinuation of study treatment were reported in two patients (6.9%; immune-mediated hepatitis in one patient and anemia, decreased platelet count, decreased white blood cell count, increased alanine aminotransferase, increased aspartate aminotransferase, and increased γ-glutamyltransferase in the other patient) in the camrelizumab + apatinib 500 mg + fuzuloparib group, and no patients discontinued study treatment due to adverse events in the camrelizumab + apatinib 375 mg + fuzuloparib group. Adverse events led to dose modification in no patients with camrelizumab + apatinib 375 mg + fuzuloparib and four patients with camrelizumab + apatinib 500 mg + fuzuloparib (13.8%; decreased white blood cell count [*n* = 2], increased γ-glutamyltransferase, decreased neutrophil count, and left ventricular dysfunction [*n* = 1 each]). Dose interruption because of adverse events occurred in one (33.3%) and 13 patients (44.8%) in the two groups, respectively (Additional file 1: Table S1). One patient (3.4%) in the camrelizumab + apatinib 500 mg + fuzuloparib group died, but the cause of death was unknown, which was deemed possibly related to the study treatment. Immune-mediated adverse events were recorded in one patient (33.3%) with camrelizumab + apatinib 375 mg + fuzuloparib and 15 patients (51.7%) with camrelizumab + apatinib 500 mg + fuzuloparib (Additional file 1: Table S2). Grade 3 or higher immune-mediated adverse events occurred in 0 and three patients (10.3%; immune-mediated hepatitis and death [*n* = 1], immune-mediated dermatitis [*n* = 1], drug-induced liver injury [*n* = 1]), respectively.

**Table 2:** Treatment-related adverse events occurring in at least 10% of patients in either group

Preferred term	Camrelizumab + fuzuloparib + apatinib 375 mg (*n* = 3)		Camrelizumab + fuzuloparib + apatinib 500 mg (*n* = 29)	
	**All grade**	**Grade ≥ 3**	**All grade**	**Grade ≥ 3**
White blood cell count decreased	2 (66.7)	0	17 (58.6)	6 (20.7)
Neutrophil count decreased	1 (33.3)	0	15 (51.7)	3 (10.3)
Platelet count decreased	0	0	13 (44.8)	2 (6.9)
Aspartate aminotransferase increased	2 (66.7)	0	12 (41.4)	3 (10.3)
Hypertension	0	0	12 (41.4)	4 (13.8)
Alanine aminotransferase increased	2 (66.7)	0	10 (34.5)	1 (3.4)
Blood bilirubin increased	0	0	9 (31.0)	0
Nausea	1 (33.3)	0	8 (27.6)	0
Asthenia	1 (33.3)	0	8 (27.6)	0
Hypothyroidism	1 (33.3)	0	7 (24.1)	0
Anemia	0	0	7 (24.1)	2 (6.9)
Blood pressure increased	1 (33.3)	0	6 (20.7)	1 (3.4)
Vomiting	2 (66.7)	1 (33.3)	5 (17.2)	0
Diarrhea	1 (33.3)	0	4 (13.8)	0
Blood creatinine increased	0	0	4 (13.8)	0
Blood lactate dehydrogenase increased	0	0	4 (13.8)	0
Sinus tachycardia	0	0	4 (13.8)	0
Gamma-glutamyltransferase increased	1 (33.3)	0	3 (10.3)	1 (3.4)
Blood thyroid stimulating hormone increased	0	0	3 (10.3)	0
Blood creatine phosphokinase increased	0	0	3 (10.3)	0
Tri-iodothyronine free decreased	0	0	3 (10.3)	0
Decreased appetite	1 (33.3)	0	3 (10.3)	0
Rash	0	0	3 (10.3)	0
Proteinuria	2 (66.7)	0	1 (3.4)	0
Hypersensitivity	1 (33.3)	0	1 (3.4)	0
Tri-iodothyronine decreased	1 (33.3)	0	1 (3.4)	0
Infusion related reaction	1 (33.3)	0	0	0
Nasopharyngitis	1 (33.3)	0	0	0
Eczema	1 (33.3)	0	0	0

Data are *n* (%)

### Efficacy

**Fig. 1:**
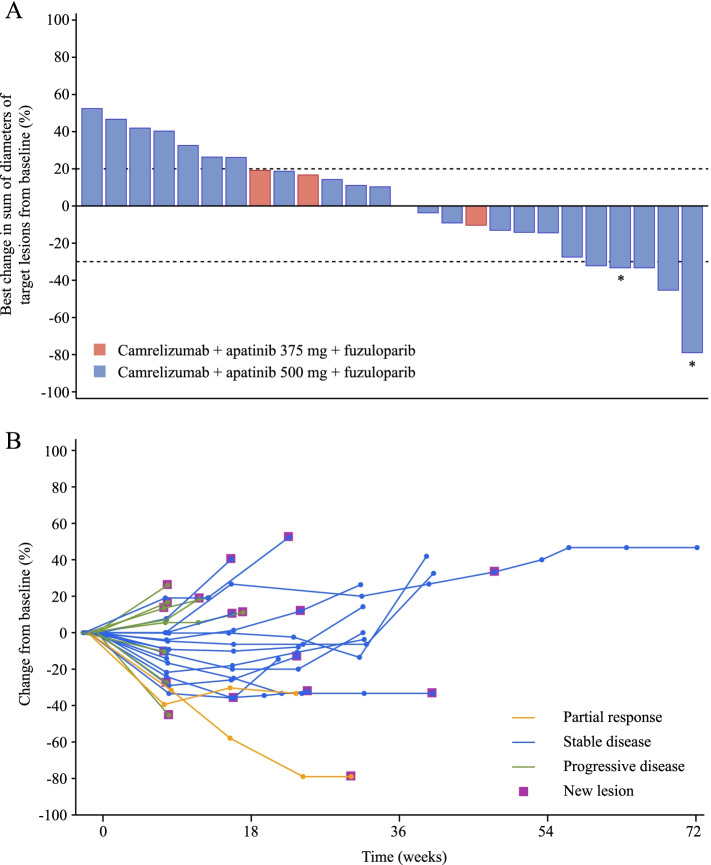
Tumor response. **A** Best percentage change in sum of diameters of the target lesion from baseline in individual patients. **B** Treatment duration and tumor response. Asterisk (*) symbol represents patients with a partial response

**Fig. 2:**
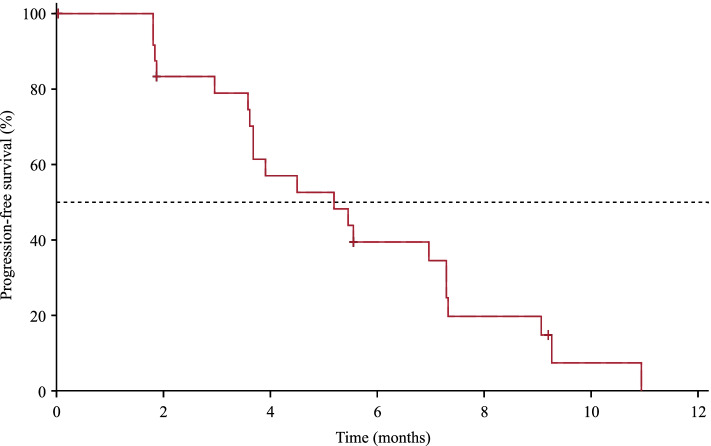
Kaplan–Meier curve for progression-free survival in patients with camrelizumab plus apatinib 500 mg and fuzuloparib

All 29 patients who received camrelizumab + apatinib 500 mg + fuzuloparib were included in the efficacy analysis set. At data cutoff, two (6.9% [95% CI, 0.9–22.8]) of 29 patients had confirmed objective responses. The study did not meet the criteria for further enrollment, and recruitment was halted. Changes in tumor burden are shown in Fig. 1A. At data cutoff, one responder had a duration of response lasting 5.1 months, and the other response lasted 3.7 months and remained ongoing. The best percentage reductions in tumor size from baseline of the two responders were 79% and 39%, respectively (Fig. 1B). Two additional responses were unconfirmed, and the unconfirmed objective response rate was 13.8% (95% CI, 3.9–31.7). Sixteen patients had stable disease, and the DCR reached 62.1% (95% CI, 42.3–79.3). The median PFS was 5.2 months (95% CI, 3.6–7.3) (Fig. 2). Long-lasting PFS was observed in two patients, with one PFS lasting 20.4 months and the other lasting 13.0 months. Five patients died, and the median OS was not reached. The 12-month OS rate was 64.2% (95% CI, 19.0–88.8). One patient in the camrelizumab + apatinib 500 mg + fuzuloparib group had germline *BRCA1* mutations, and the best overall response was stable disease.

### Pharmacokinetics

The pharmacokinetic parameter analysis demonstrated that the absorption of apatinib was fast, with a median *t*_max_ of 2.0 h both after single dosing (day − 3 of cycle 0) and combined dosing with fuzuloparib (day 1 of cycle 1). The median *t*_max_ for fuzuloparib was 3.0 h. Apatinib exposure (*C*_max_ and AUC_0-24_) increased as the dose level increased from 375 to 500 mg after both single-agent dosing and combined administration with fuzuloparib (Table 3). Fuzuloparib exposure in terms of *C*_max_ and AUC_0-24_ was similar between groups.

**Table 3:** Pharmacokinetic parameters of apatinib and fuzuloparib on day − 3 of cycle 0 and day 1 of cycle 1

	**Apatinib**		**Fuzuloparib**	
	**Camrelizumab + fuzuloparib + apatinib 375 mg (*n* = 3)**	**Camrelizumab + fuzuloparib + apatinib 500 mg (*n* = 8)**	**Camrelizumab + fuzuloparib + apatinib 375 mg (*n* = 3)**	**Camrelizumab + fuzuloparib + apatinib 500 mg (*n* = 8)**
**Day − 3 of cycle 0**				
*C*_max_, ng/mL	291 (80.4)	576 (362)	**—**	**—**
*t*_max_, h	2.0 (2.0–8.0)	2.0 (1.0–8.0)	**—**	**—**
AUC_0-24/0–12_, h x ng/mL^a^	2710 (109)	4120 (2140)	**—**	**—**
**Day 1 of cycle 1**				
*C*_max_, ng/mL	420 (340)	709 (601)	2720 (348)	3740 (1770)
*t*_max_, h	2.0 (1.0–8.0)	2.0 (1.0–8.0)	3.0 (2.0–3.0)	3.0 (2.0–7.9)
AUC_0-24/0–12_, h x ng/mL^a^	1510 (1630)	4220 (2730)	21,200 (2330)	25,800 (17,900)

^a^For apatinib, AUC_0-24_ was reported; for fuzuloparib, AUC_0-12_ was reported. Data are mean (standard deviation) or median (range)

*Abbreviations*: *C*_*max*_ maximum plasma concentration, *t*_*max*_ time to *C*_max_, *AUC* area under the plasma concentration time curve

## Discussion

This phase Ib study aimed to investigate the tolerability, safety, preliminary antitumor activity, and pharmacokinetic parameters of camrelizumab plus apatinib and fuzuloparib in patients with recurrent or metastatic TNBC. To our knowledge, this is the first reported study to evaluate the combined blockade of PD-1, VEGFR, and PARP for TNBC. Among the two dosing regimens in the dose-finding part, no DLTs were reported, and camrelizumab 200 mg + apatinib 500 mg + fuzuloparib 100 mg was established as the clinical recommended dose.

The adverse events in our study were generally acceptable. In a previous phase II trial, camrelizumab 200 mg every 2 weeks combined with apatinib 250 mg once daily showed a manageable safety profile in patients with advanced TNBC [13]. In the present study, an escalated dose of apatinib (500 mg vs. 250 mg) and the addition of fuzuloparib elevated the risk of grade ≥ 3 TRAEs (58.6% vs. 26.7%) compared with reports from the phase II trial. However, the TRAE profile was similar between the two trials, with no new safety signals observed [13]. In addition, only two patients discontinued study treatment due to adverse events in the camrelizumab + apatinib 500 mg + fuzuloparib group. Most of the TRAEs of grade 3 or higher were hematological adverse events, with the most frequently reported ones being decreased white blood cell count, hypertension, decreased neutrophil count, and increased aspartate aminotransferase [13]. Notably, hand-foot syndrome and proteinuria, which were among the most common TRAEs of apatinib, had a low incidence in our trial. The underlying mechanism is unknown, and this finding requires future investigation.

TNBC has a poor prognosis in the recurrent or metastatic setting [2]. Immune checkpoint inhibitors alone show limited efficacy [22]. Therefore, combination strategies urgently need to be developed to improve the therapeutic outcomes of TNBC. PARP inhibitor-mediated dysfunction in DNA damage repair may result in increased mutation load and neoantigen burden, which can increase PD-L1 expression and render tumor cells response to immune checkpoint inhibitors [23, 24]. Therefore, combining PARP inhibitors with immune checkpoint inhibitors seems to be a reasonable approach. In the phase I/II MEDIOLA trial, olaparib plus durvalumab showed a DCR of 80% at week 12 and 50% at week 28 and an ORR of 63% at week 12 in patients with germline *BRCA*-mutated metastatic HER2-negative breast cancer [25]. In the phase II TOPACIO trial, niraparib plus pembrolizumab showed clinical benefit (ORR, 21%; DCR, 49%; median PFS, 2.3 months) in patients with advanced or metastatic TNBC irrespective of *BRCA* mutation status, although the antitumor activity was better in patients with *BRCA* mutation than in those with *BRCA* wild-type tumors (ORR, 47% vs. 11%; DCR, 80% vs. 33%; median PFS, 8.3 vs. 2.1 months) [26]. Mounting evidence has confirmed the antitumor activity of PARP inhibitors plus anti-PD-1/PD-L1 antibodies in various types of cancers.

Based on the preclinical rationale, we expected that the combination of a VEGFR inhibitor with a PARP inhibitor and an immune checkpoint inhibitor would further modulate the tumor microenvironment and activate antitumor immunity. However, this combination showed limited antitumor activity in patients with recurrent or metastatic TNBC in the present study. Two of the 29 patients with camrelizumab + apatinib 500 mg + fuzuloparib had an objective response. In contrast to our results, the combination of camrelizumab 200 mg every 2 weeks with apatinib 250 mg once daily showed an ORR of 43.3% (95% CI, 25.5–62.6) [13]. Previous research showed that a low dose of anti-VEGF therapy resulted in vascular normalization and improved antitumor immunity, while a high dose induced hypoxia and an immunosuppressive tumor microenvironment [11, 27, 28]. The low response rate in our study might be attributed to the high dose level of apatinib. However, we observed a comparable DCR (63.3% vs. 62.1%), longer median PFS (5.2 months vs. 3.7 months), and higher 12-month OS rate (64.2% vs. 42.2%) between our study and the study with camrelizumab and low dose apatinib in patients with advanced TNBC [13]. However, these data should be interpreted cautiously given the difference in patient selection and study design. Future studies are warranted to investigate the optimal dosing regimen of this combination and better understand the molecular mechanisms.

In the present study, apatinib exposure increased with increasing doses, which was consistent with previous reports [29]. Apatinib exposure was comparable before and after co-administration with fuzuloparib. However, due to the small sample size, future investigation is needed to further confirm this conclusion.

The major limitation of the study is the lack of biomarker analysis. Identification of biomarkers is important for treatment strategy development. A recently published biomarker analysis study showed that famitinib (an angiogenesis inhibitor) plus camrelizumab and chemotherapy had an impressive clinical benefit in patients with CD8-positive advanced TNBC, with an ORR of 81.3% and a median PFS of 13.6 months, and those with CD8- and PD-L1-positive tumors benefit more from this regimen [30]. However, combining anti-PD-1/PD-L1 antibody with anti-angiogenesis agent showed clinical benefit irrespective of PD-L1 expression in several other tumor types [31–33]. The prognostic value of PD-L1 in patients with advanced TNBC who received immunotherapy plus anti-angiogenesis needs further investigation. In addition, *BRCA*-mutant tumors are more likely to respond to PARP inhibitors in combination with immune checkpoint inhibitors [26]. However, the small number (only one patient) of *BRCA*-mutant patients in our study precluded firm conclusions about the efficacy of PARP inhibitor in *BRCA*-mutant patients. Future studies with biomarker analysis are warranted to identify patients who would benefit most from this combination regimen.

## Conclusions

Camrelizumab plus apatinib and fuzuloparib showed manageable safety profile in patients with recurrent or metastatic TNBC. Although the ORR was low in this study, the DCR and PFS were promising. Future studies are needed to optimize the dosing regimen of this combination therapy.

## Supplementary Information

**Additional file 1:** **Table S1.** Adverse events leading to dose interruption in either group. **Table 2.** Immune-mediated adverse events in either group.

## Data Availability

The data that support the findings of this study are available from the corresponding author upon reasonable request.

## Abbreviations

TNBC: Triple-negative breast cancer
EG: Estrogen receptor
PR: Progesterone receptor
HER2: Human epidermal growth factor receptor 2
OS: Overall survival
PD-1: Programmed cell death-1
VEGFR-2: Vascular endothelial growth factor receptor-2
ORR: Objective response rate
PARP-1: Poly (ADP-ribose) polymerase-1
IHC: Immunohistochemistry
ECOG: Eastern Cooperative Oncology Group
RECIST: Response Evaluation Criteria in Solid Tumors
DLT: Dose-limiting toxicity
CT: Computed tomography
MRI: Magnetic resonance imaging
DOR: Duration of response
DCR: Disease control rate
PFS: Progression-free survival
*C*_max_: Maximum plasma concentration
*t*_max_: Time to *C*_max_
AUC: Area under the plasma concentration time curve
CI: Confidence interval
TRAEs: Treatment-related adverse events
