# Supplementary material for: A phase Ib study of camrelizumab in combination with apatinib and fuzuloparib in patients with recurrent or metastatic triple-negative breast cancer
Source: BMC Med. 2022 Oct 3;20:321. doi: 10.1186/s12916-022-02527-6 (PMC9528096; doi:10.1186/s12916-022-02527-6)
Supplement: Supplementary file 1 — Additional file 1: Table S1. Adverse events leading to dose interruption in either group. Table 2. Immune-mediated adverse events in either group. [file 12916_2022_2527_MOESM1_ESM.docx]

Supplementary Material

**Content**

[**Table S1.** Adverse events leading to dose interruption in either group 2](#_Toc103260328)

[**Table S2.** Immune-mediated adverse events in either group 3](#_Toc103260329)

# **Table S1.** Adverse events leading to dose interruption in either group

| **Terms** | **Camrelizumab + fuzuloparib +**  **apatinib 375 mg (n = 3)** | **Camrelizumab + fuzuloparib +**  **apatinib 500 mg (n = 29)** |
| --- | --- | --- |
| White blood cell count decreased | 0 | 7 (24.1) |
| Neutrophil count decreased | 0 | 5 (17.2) |
| Aspartate aminotransferase increased | 0 | 3 (10.3) |
| Platelet count decreased | 0 | 3 (10.3) |
| Alanine aminotransferase increased | 0 | 2 (6.9) |
| Anaemia | 0 | 2 (6.9) |
| γ-glutamyltransferase increased | 0 | 2 (6.9) |
| Vomiting | 1 (33.3) | 1 (3.4) |
| Drug-induced liver injury | 0 | 1 (3.4) |
| Dyspnoea | 0 | 1 (3.4) |
| Hepatic function abnormal | 0 | 1 (3.4) |
| Hypersensitivity | 0 | 1 (3.4) |
| Immune-mediated dermatitis | 0 | 1 (3.4) |
| Immune-mediated hepatitis | 0 | 1 (3.4) |
| Left ventricular dysfunction | 0 | 1 (3.4) |
| Lymphocyte count decreased | 0 | 1 (3.4) |
| Nausea | 0 | 1 (3.4) |
| Pneumonia | 0 | 1 (3.4) |
| Pyrexia | 0 | 1 (3.4) |
| Upper respiratory tract infection | 0 | 1 (3.4) |

Data are n (%).

# **Table S2.** Immune-mediated adverse events in either group

| **Terms** | **Camrelizumab + fuzuloparib +**  **apatinib 375 mg (n = 3)** | | **Camrelizumab + fuzuloparib +**  **apatinib 500 mg (n = 29)** | |
| --- | --- | --- | --- | --- |
|  | **All grade** | **Grade ≥3** | **All grade** | **Grade ≥3** |
| Hypothyroidism | 1 (33.3) | 0 | 5 (17.2) | 0 |
| White blood cell count decreased | 0 | 0 | 3 (10.3) | 0 |
| Blood thyroid stimulating hormone increased | 0 | 0 | 2 (6.9) | 0 |
| Hyperthyroidism | 0 | 0 | 2 (6.9) | 0 |
| Alanine aminotransferase increased | 0 | 0 | 1 (3.4) | 0 |
| Arthralgia | 0 | 0 | 1 (3.4) | 0 |
| Aspartate aminotransferase increased | 0 | 0 | 1 (3.4) | 0 |
| Blood creatine phosphokinase increased | 0 | 0 | 1 (3.4) | 0 |
| Blood creatine phosphokinase MB increased | 0 | 0 | 1 (3.4) | 0 |
| Blood pressure increased | 0 | 0 | 1 (3.4) | 0 |
| Death | 0 | 0 | 1 (3.4) | 1 (3.4) |
| Decreased appetite | 0 | 0 | 1 (3.4) | 0 |
| Drug-induced liver injury | 0 | 0 | 1 (3.4) | 1 (3.4) |
| Electrocardiogram ST-T change | 0 | 0 | 1 (3.4) | 0 |
| Fatigue | 0 | 0 | 1 (3.4) | 0 |
| Flank pain | 0 | 0 | 1 (3.4) | 0 |
| Hypertension | 0 | 0 | 1 (3.4) | 0 |
| Immune-mediated dermatitis | 0 | 0 | 1 (3.4) | 1 (3.4) |
| Immune-mediated hepatitis | 0 | 0 | 1 (3.4) | 1 (3.4) |
| Immune-mediated hypothyroidism | 0 | 0 | 1 (3.4) | 0 |
| Left ventricular dysfunction | 0 | 0 | 1 (3.4) | 0 |
| Nausea | 0 | 0 | 1 (3.4) | 0 |
| QRS axis abnormal | 0 | 0 | 1 (3.4) | 0 |
| Rash | 0 | 0 | 1 (3.4) | 0 |
| Sinus tachycardia | 0 | 0 | 1 (3.4) | 0 |
| Thyroid function test abnormal | 0 | 0 | 1 (3.4) | 0 |
| Tri-iodothyronine free decreased | 0 | 0 | 1 (3.4) | 0 |
| Vomiting | 0 | 0 | 1 (3.4) | 0 |
| γ-glutamyltransferase increased | 0 | 0 | 1 (3.4) | 0 |

Data are n (%).
